# Young dispersal of xerophil *Nitraria* lineages in intercontinental disjunctions of the Old World

**DOI:** 10.1038/srep13840

**Published:** 2015-09-07

**Authors:** Ming-Li Zhang, Kamshat Temirbayeva, Stewart C. Sanderson, Xi Chen

**Affiliations:** 1 Key Laboratory of Biogeography and Bioresource in Arid Land, Xinjiang Institute of Ecology and Geography, Chinese Academy of Sciences, Urumqi 830011, China; 2 Institute of Botany, Chinese Academy of Sciences, Beijing 100093, China; 3 Shrub Sciences Laboratory, Intermountain Research Station, Forest Service, U.S. Department of Agriculture, Utah 84601, USA

## Abstract

Many cases of intercontinental disjunct distributions of seed plants have been investigated, however few have concerned the continents of Eurasia (mainly Central Asia), Africa, and Australia, especially the xerophytic lineages are lacking. *Nitraria* (Nitrariaceae) is just one of these xerophytic lineages. Previous *Nitraria* studies have hypothesized either Africa as the ancient center, with dispersals to Australia and Eurasia, or alternatively Central Asia, due to a concentration of endemism and diversity there. Our findings show eastern Central Asia, i.e. the eastern Tethys, to be the correct place of origin. Dispersal westward to Africa occurred during the late Oligocene to Pliocene, whereas dispersal to Australia from western Central Asia was young since Pliocene 2.61 Ma. Two related tetraploids are indicated to have diversified in eastern Central Asia at approximately 5.89 Ma, while the Australian tetraploid *N. billardieri*, is an independently derived, recent dispersal from western Central Asia.

Intercontinental disjunct distribution is always an attractive topic for plant biogeographers^1^. Thorne^2^ concluded that the largest number of disjunct seed plant genera are found with an African - Eurasian (Pacific) disjunction (600), then orderly Asian—Pacific (460), Pacific (370), North American-South American (ca. 360), Pantropical (334), North Temperate (316), etc. Wen *et al*.^3^ reviewed that typical disjunctions comprise three patterns, i.e. northern hemisphere, pantropical, and southern hemisphere. One example in Northern Hemisphere is famous Asa Gray’s East Asia - North America disjunction, much research has dealt with it.Intercontinental disjunct distributions can be explained either by vicariance related to plate tectonics, or by long distance transoceanic dispersal^4^,^5^,^6^. It has always been related to paleogeographic events, and its study has been considerably promoted by the analytical approaches of recent decades, such as molecular clock and ancestral area reconstruction^3^.

Although many intercontinental disjunctions have been examined, nevertheless, few plant genera are comparable to *Nitraria*, in regard to its Africa - Central Asia - Australia disjunction, especially it as one of xerophytic lineages. *Nitraria* was previously placed in Zygophyllaceae, but now is included in Nitrariaceae^7^,^8^. It consists of 8(10) to 12 species occurring in arid lands, saline grasslands, and sandy deserts, see Fig. 1. These shrubs are used in protective afforestation for stabilizing sand deposits and banks, reduction of soil salinity, augmentation of soil organic matter, and medicine in northwestern China^8^,^9^,^10^. Its salty-sweet edible fruits are considered an important food for animals, therefore, its dispersal relies on birds, mammals, and occasionally ants^9^,^10^,^11^.

Komarov^9^ and Bobrov^12^ revised taxonomy of the genus. Systematic evidence has accumulated from pollen morphology^13^,^14^, chromosomes^15^,^16^, and molecular phylogeny^17^. Based on morphology and pollen characters, Pan *et al*.^14^ established three sections in *Nitraria*. Chromosome evidence shows that there are diploid (2n = 24) and tetraploid (2n = 48) species in the genus, therefore, *Nitraria* is divided into two groups of diploid and tetraploid^18^. Recently, molecular phylogeny using ITS and cpDNA sequences has improved the generic classification and species relationship^17^.

Komarov^9^ hypothesized *Nitraria* to have originated from Africa, with dispersals to Europe and Australia. And he supposed these to have been ancient events, occurring before separation of the African and Australian plates from Gondwana, which would mean before at least 50 Ma; Vassilchenko^19^ and Pan *et al*.^14^ were in agreement with him. Based on the distribution of diploid species, Pan *et al*.^18^ speculated an African-Mediterranean origin. Yang^20^ also thought an African origin likely, however, dispersal to Australia was assumed to have been a migration via Asia. In contrast to the African origin, Bobrov^12^ assumed that the Cretaceous and Palaeogene plateaus, which were once covered with forest and became the ancient Central Asian desert, were the likely ancestral area. Grubov^21^ also proposed the genus to be native to Central Asia, because the area is rich in endemic species and had ancient transformative geological events.

To test these biogeographical hypotheses, the robust methodology of molecular clock and ancestral areas reconstruction, developed in the last decades, are used to investigate *Nitraria* origin and evolution, and reconstruct the biogeographical history of *Nitraria* disjunct distribution and speciation.

## Results

### Molecular phylogenetic tree and dating

In Fig. 2, BEAST dating results of the 7-gene dataset for stem and crown ages of *Nitraria* were respectively 85.3 Ma (95% HPD 70.55–103.59) (48.66 Ma, *rbc*L dating, see Supporting Information S2) and 61.84 Ma (95% HPD 57.7–75.26) (33.5 Ma, *rbc*L dating). The *rbc*L dating results were smaller than those of the 7-gene dating, and exceptionally *N. sphaerocarpa* was out of *Nitraria* and nested in *Peganum*. The dating result shows that *Nitraria* is in reality an ancient taxon, originating in early Paleocene to Cretaceous, yet diversification within *Nitraria* is dated to late Miocene 8.96 Ma foreward. Except for *N. sphaerocarpa* and African *N*. *retusa*, most of the species nodes or clades of the genus are young, less than 9 Ma, late Miocene to Pliocene.

### Ancestral area reconstructions

Since many small, uncertain probability events appeared at nodes of the tree using Langrage, its result was ignored. Ancestral area reconstruction employing S-DIVA and BBM (Fig. 3) in RASP v2.1, having roughly similar results at the nodes, indicated that the ancestral area for *Nitraria* was eastern Central Asia (A), that the MRCA area of the African *N. retusa* was eastern Central Asia (A), and that the MRCA of Australian *N. billardieri* was in western Central Asia (B). Dispersals estimated from S-DIVA and BBM (Fig. 3) are shown in Fig. 3.

Four dispersals occurred in *Nitraria*. One was to Africa (D) from eastern Central Asia (A), one was from eastern (A) to western Central Asia (B), and one was to Siberia (C) from Central Asia (AB), and one was to Australia (E) from western Central Asia (B). We also label these dispersals with temporal dating (Fig. 2), to plot evolutionary events.

### Diversification rate

LTT plot is shown in Fig. 2, which clearly indicates an increase of the diversification rate (accelerated lineage accumulation) within *Nitraira* after ca. 8.96 Ma in the late Miocene and towards the current time (Fig. 2). The yule3rate model, which includes two diversification rate shifts and no extinction, was found to fit the data best (AIC = 39.4691), it is shown that the data set better fits a rate-variable model.

By calculating the net diversification rates of *Nitraria* and its internal clades, we found that some lineages during the late Miocene had an elevated diversification rate, such as nodes 13, 15, and 16, see Support Information S3. The absolute diversification rates (r) estimated for clades of *Nitraria*, considering both no extinction (ε = 0.0) and a high relative extinction rate (ε = 0.9), are shown. The average diversification rates estimated for the crowns of nodes 13, 15 and 16 (r_0.0_ = 0.200, 0.233, 0.262 speciation events per million years (sp Myr^−1^) for ε = 0, and r_0.9_ = 0.045, 0.044, 0.044 sp My^−1^ for ε = 0.9) are considerably higher than those estimated for other nodes within *Nitraria*.

### Chromosome evolution

Even though many hypotheses can be detected by the chromosome evolution program, in *Nitraria* the situation is simple, with only a duplication from diploid to tetraploid, and with diploids estimated at most nodes of the *Nitraria* BEAST tree. Two tetraploid “gain” events are indicated, one at the node of the MRCA of *N. roborowiskii* and *N. tangutorum*, and the other at the *N. billardieri* branch, the latter with probabilities near 1.0, as labeled in Fig. 2.

## Discussion

### Ancestral area

Based on the dating chronogram (Fig. 2), the ancestral area reconstruction for *Nitraria* clearly shows eastern Central Asia as the ancestral area (Fig. 3). It is therefore not in agreement with hypotheses of an African origin, such as that of Komarov^9^ and followers^14^,^18^,^19^,^20^, but prefers Bobrov^12^ and Grubov^21^, who stressed the antiquity of the Mongolian to Central Asian desert flora. In addition, Komarov’s^9^ suggestion of the Mongolian flora as being young seems implausible from our *Nitraria* evidence.

We can demonstrate the ancestrality of eastern Central Asia as follows. *Nitraria* has an estimated stem age of 85.3 (95% HPD 70.55–103.59) (48.66 Ma, *rbc*L dating) and a crown age of 61.84 (95% HPD 57.7–75.26) Ma (33.5 Ma, *rbc*L dating) (see Fig. 2). Eastern Central Asia, located along the eastern Tethys^22^, was an ancient land that in early Tertiary, from Paleocene to Oligocene, had an arid subtropical flora and vegetation^23^ which could have fostered the growth and development of ancient arid lineages such as *Nitraria*. Antiquity of the eastern Central Asian flora can be evidenced from pollen complexes in several regions such as Kashgar, the Zaidam Basin, and the Hexi Corridor in China^24^,^25^,^26^,^27^, particularly *Nitraria* pollen found in Paleocene strata at Keche county, Xinjiang province (located on the southern slope of the Tianshan Mts. and pertaining to the Kashgar flora)^13^; and the late Eocene (Paleocene-Cretaceous) at Xining, Qinghai province^28^, having old arid vegetation^28^,^29^, and arid endemic genera^30^. Eastern Central Asia could be regarded as the ancestral area because of the richness of *Nitraria* species there, with six endemic species, and with *N. sphaerocarpa* being located at a basal position on the phylogenetic tree (Fig. 2).

### Dispersals and their driving causes

According to our biogeographical analysis (Fig. 3), four dispersals occurred in the evolutionary history of *Nitraria*. One was from eastern Central Asia (A) to Africa (D) during 30-2.63 Ma, the second from eastern Central Asia (A) to western Central Asia (B) during 8.96-5.95 Ma, the third from Central Asia (AB) to Siberia since 5.95 Ma and the last from western Central Asia (B) to Australia (E) since 2.61 Ma (Figs 2 and 3). In the light of these spatiotemporal events, we can trace and depict causes of the dispersals.

First, since four dispersals appeared in the Oligocene to late Tertiary, according to tectonic history^22^, the migrations among Central Asia, northern Africa and Australia, might have been possible mostly via land, see the three tectonic relief maps (Fig. 2). Cases of plant dispersal by overland expansion have been elucidated from Asia to Africa via the Arabian peninsula, such as *Macaranga* (Euphorbiaceae)^31^, and *Isodon* (Lamiaceae)^6^, whereas the terrestrial and aquatic family Haloragaceae was shown to disperse from Australia to Asia - Europe - North America, during the Miocene (16 Ma) to Pleiocene^32^. Our early dispersal pattern since 30 Ma might therefore contrast with the ancient trans-oceanic rafting hypothesis (e.g. Cretaceous^33^), or trans-oceanic long-distance dispersal^4^,^34^.

**Figure 1:**
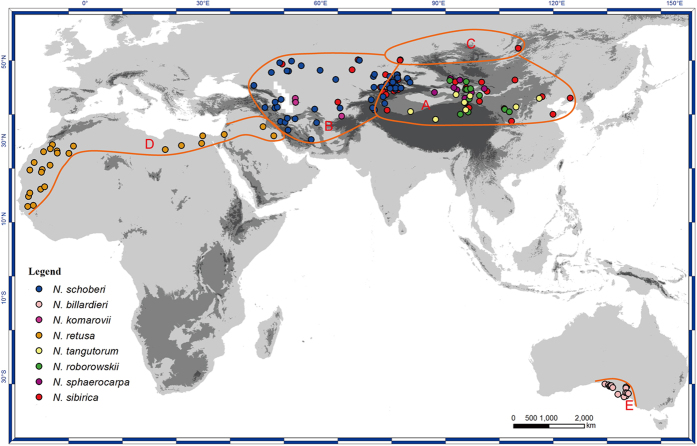
*Nitraria* distribution, with five areas in the Old World, distribution spots data are come from our recorded specimens in herbaria and the floras, distribution range of some species is referred Noble & Whalley^14^, and Pan *et al*.^19^. Basemap is come from WorldClim Database (http://www.worldclim.org/).

**Figure 2:**
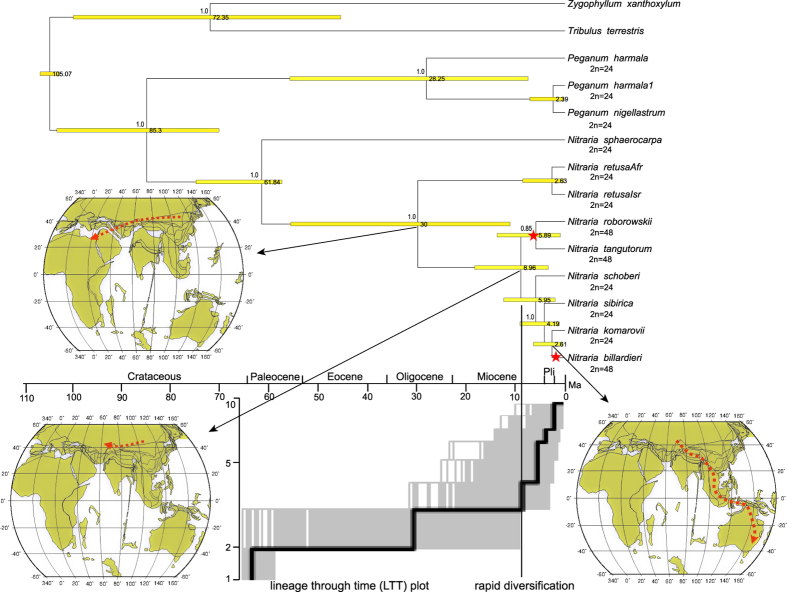
Chronogram of *Nitraria* and outgroups, with maximum clade credibility, performed by BEAST. Area label letters are the same as for Fig. 1. Dating values are plotted at the right of the nodes, and posterior probability support values are labeled at left above the nodes. Inset are three paleomap globes showing temporal and spatial dispersals with red dashed-line arrows. Three paleomaps come from http://www.odsn.de/odsn/services/paleomap/paleomap.html. The LTT results show a rapid diversification at 8.96 Ma. Chromosome evolution and two duplication (tetraploid) events, labeled with red stars, are shown respectively at the node of the MRCA of *N. robovoviskyi* and *N. tangutorum*, and at the branch of *N. billardieri*.

**Figure 3:**
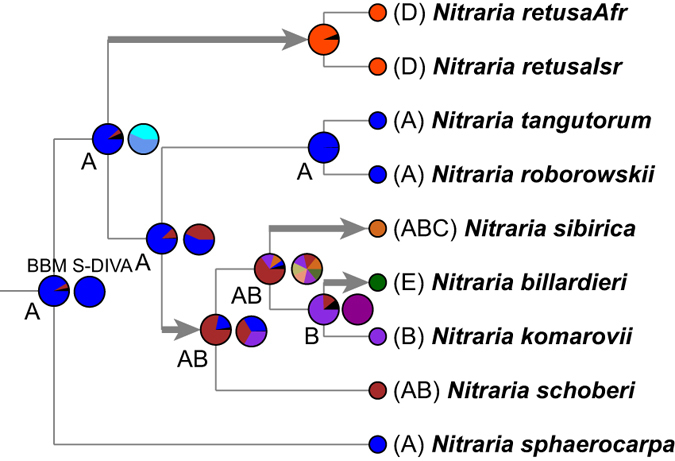
Biogeographical ancestral optimization performed with S-DIVA and BBM, implemented in RASP. Pie charts at the internal nodes represent the calculated probabilities (relative frequencies) of alternative ancestral areas (reconstructions), as produced by BBM and S-DIVA (right). Four dispersals are shown, with thick- lined arrows for the branches. Below, curves of dispersal are indicated through time. Area letters are the same as in Fig. 1, and details are stated in the text.

*Nitraria retusa* in northern Africa and adjacent regions is indicated to be a dispersal from eastern Central Asia during 30-2.63 Ma, see Figs 2 and 3. In terms of its extant distribution (Fig. 1), this dispersal may have been associated with the western retreat and closing of the Tethys Sea, which occurred at the boundary of the Oligocene and early Miocene, with final closure at ca. 20 Ma^35^,^36^,^37^. The African and Eurasian plates had a convergence during the Oligocene (34-30 Ma), the cause of the retreat of the Tethys^22^, which raised massive land areas, perhaps offering a possibility of overland dispersal of *Nitraria* to Africa. The ancestor of *Nitraria*, indicated by ancestral area reconstruction (Fig. 3), inhabited the coastal eastern Tethys, from where its dispersal might have begun as shown in Fig. 2.

Rapid diversification of *Nitraria* since ca. 8.96 Ma is obvious using LTT in Fig. 2. It occurred mainly in Central Asia, as seen events in Figs 2 and 3, the rich diversification of species, several clades born in Central Asia, several dispersals from eastern into western Central Asia during ca. 8.96–5.95 Ma, as well as the origin of polyploidy within eastern Central Asia (with tetraploid *N. roborowskii* and *N. tangutorum*). We can presume that the rapid diversification was most likely associated with expansion of the QTP and global climate cooling and drying since ca. 10-8 Ma^38^,^39^. This is because the diversification of *Nitraria* temporally fits the event, as well as augmented aridification intensity in Central Asia at ca. 10-8 Ma, which drove by QTP rapid uplift. These geological and climate factors most likely drove rapid diversification of arid lineages, as has been evidenced by many plant cases, such as *Caragana*^40^, *Artemisia*^41^, *Myricaria*^42^, *Atraphaxis*^43^, and *Reaumuria*^44^. On the other hand, our inferred dispersal events within Central Asia during 8.96-5.95 Ma (Figs 2 and 3), are also responses to these events; the union of arid climate and vegetation in eastern and western Central Asia during this period generated the possibility of spatiotemporal dispersals^23^,^41^,^45^. Dispersal from Central Asia northward to adjacent Siberia was also possible.

The difference in diversification ages of the African and Australian taxa illuminates the equivocality of the idea of a disjunction between Africa and Australia. The phylogenetic tree (Fig. 2) indicates that the Australian species *N. billardieri* represents a young dispersal event occurring in the Pliocene at 2.61 Ma and nested in the mass of the Central Asian species *N. komarovii, N. sibirica*, and *N. schoberi* (Fig. 3). This age rules out the vicariance hypothesis involving the Gondwana separation between Africa and Australia by Komarov^9^, whereas it may suggest the possibility of an overland dispersal, along the Himalayas, India, Myanmar, and Malaysia to Australia from western Central Asia, in accordance with Yang^20^. However, the predominance of incompatible monsoon forest vegetation in most of southeast Asia, seems to largely rule out this alternative for an arid-adapted species^46^.

A more felicitous possibility seems to be transoceanic long distance dispersal due to the characteristic of *Nitraria* berry fruits and the consumption of these by birds^10^. Although there is a regular flyway for shorebirds from East Asia and Alaska to Australia^47^,^48^, there are not any regular migrations going to Australia from Central Asia. This is likely because of a long over-water distance and the presence of contrary easterly trade winds during the time for fall migration^46^,^49^. Nevertheless, the Caspian plover or dotterel (mostly insectivorous), normally migrates to South Africa from Central Asia, and is recorded rarely in Australia^50^. An assortment of Asian bird species might be expected to appear occasionally in Australia in this manner, which over geologic time would possibly have provided sufficient opportunity for dispersal of seeds of *Nitraria*.

### Polyploidy

Polyploidy in *Nitraria* is simple, with only diploid (2n = 24) and tetraploid (2n = 48) species, the tetraploid should be a simple duplication from diploid from our analysis (Fig. 2). The tetraploid species display remarkable implications for the generic evolutionary history. Tetraploidy Australian *N. billardieri* suggests it to be a derived species, in agreement with the above phylogenetic conclusion, with a young estimated stem age of only 2.61 Ma. Two other tetraploid species, *N. roborowskii* and *N. tangutorum*, endemic to eastern Central Asia, and with relatively young crown ages of 5.89 Ma (95% HPD0.93–13.82) (Fig. 2), act as one of four clades within genus, an derived clade of the phylogenetic phase^17^. Therefore, eastern Central Asia with ancient diploid basal *N. sphaerocarpa*, and young derived tetraploid of phylogenetic phases, should be regarded as the ancestral area for *Nitraria* in terms of the critique of origin center^51^.

It is reasonable that the polyploidy of *N. roborowskii* and *N. tangutorum* in eastern Central Asia is an apparent *in situ* development from their most recent common ancestor (MRCA) in that region (Fig. 2). However, to explain *N. billardieri* dispersal in Australia, should link its close species *N. schoberi* (Fig. 2), which mainly distributed in western Central Asia. Whereas *N. billardieri* was previously even treated as a variety^9^ or synonym^52^ of *N. schoberi*. Therefore, *N. billardieri* possibly dispersed from an ancestor in Central Asia, , and it would have experienced lengthy pressures of aridification and salinity as well as poor soil and harsh environments, being coupled with woodlands and grasslands/herbfield vegetation developing in the Late Pliocene-Pleistocene in Australia^53^, became adapted and developed tetraploid descendants in southern Australia.

## Methods

### Taxon sampling and DNA sequencing

Eight species in *Nitraria* were sampled; *Peganum harmala* L. and *P. nigellastrum* Bunge in Nitrariaceae, and distant *Tribulus terrestris* L. and *Zygophyllum xanthoxylum* Maxim. in Zygophyllaceae were chosen as outgroups, justifiable because of the great age depth of the Nitrariaceae. Seven DNA fragments, including *nr*DNA ITS, *cp*DNA *rbc*L, *psb*B-*psb*H, *trn*L-*trn*F, *rp*S16, *psb*A-*trn*H, *rp*S16-*trn*K, were sequenced^17^. A dataset with 6301 bps was used for phylogenetic dating, see Supporting Information S2 Table 1.

### Molecular phylogenetic tree and dating

Bayesian phylogenetic analysis and divergence time estimates were together implemented in BEAST 1.5.4 (http://beast.bio.ed.ac.uk/). We used the uncorrelated lognormal relaxed clock model with a Yule process for the speciation model, GTR + I + G for the substitution model (estimated for the dataset).

*Nitraria* and *Peganum* are included in Nitrariaceae, whose crown age has been estimated at (96.5) 86.1 or 57.7 Ma^54^, while *Tribulus* and *Zygophyllum* are included in Zygophyllaceae. In traditional systematics, *Nitraria* and *Peganum* were also included in Zygophyllaceae (e.g. Cronquist^55^; Takhtajian^56^); Zygophyllaceae (or Zygophyllales) is estimated at 79-55 or 102 Ma^54^. A union of these four genera has been dated to 105 Ma^57^, thus, for the 7-gene dataset, 105 Ma is suitably designated here as the root age, and we used a normal prior, Mean = 105 Ma, Stdev = 1. In terms of *Nitraria* pollen fossils, there are records from the Paleocene in Xinjiang^13^, and from 60 (80) Ma in Xining^28^, as well as a compatible estimate of 86.1 or 57.7 Ma^54^, so we constrained the *Nitraria* crown age to be prior Uniform, with Lower 57.7 Ma and Upper 86.1 Ma in BEAST.

Concerning current *Nitraria* and Nitrariaceae systematics, we extended outgroups into Sapindales, related Malvales, and even Zygophyllales. We intensely sampled families and genera within Sapindales and Malvales using *rbc*L sequences downloaded from GenBank, see Supporting Information S1 Table 1. The root of all taxa sampled is the same as mentioned above, with a normal prior 105 Ma and stdev = 1. According to fossils reviews of Sapindales^54^, Simaroubaceae is assigned at 52 Ma due to fruits fossil of *Ailanthus*, and a union of *Acer* and *Aesculus* fossil fruits in Sapindaceae is shown by its 63 Ma age, while *Biebersteinia* is dated from fossil pollen to an age of 54.8 ~ 57 Ma. Thus, the root was set up as 105 Ma normal, and *Biebersteinia* with Uniform Lower = 54.8, Upper = 57, are designated, and then two constraint priors schemes are used in BEAST. Prior I, Simaroubaceae with lognormal offset = 52 Ma, mean = 0, stdev = 1; union of *Acer* and *Aesculus* with offset = 63 Ma, mean = 0, stdev = 1. Prior II, Simaroubaceae with Uniform, Lower = 52, Upper = 90 (Muellner^54^ Bayesian estimated crown age of sapindales 90 Ma), union of *Acer* and *Aesculus* Uniform Lower = 63, Upper = 90.

A Markov chain Monte Carlo was run for 50 million generations and sampled every 1,000 generations for the 7-gene dataset and *rbc*L datasets of Priors I and II. The two resulting BEAST trees of Priors I and II were combined with LogCombiner v.1.5.4. The stationarity of runs was examined using the effective sampling size of each parameter (>200). TreeAnnotator v 1.7.2 was used to summarize the post burn-in (10%) trees and their parameters. FigTree 1.3.1 was used for visualizing the final tree. The dating results for 7-gene dataset and *rbc*L dataset, are shown respectively in Fig. 2 and Supporting Information S2.

### Ancestral area reconstruction

Five areas were used in ancestral area reconstruction. Central Asia includes mainly desert and steppe regions, as well as some significant mountains. In term of floristic divisions, two parts, Mongolia and the Junggar-Turan province^21^, or the Junggar and Kashgar subkingdoms^58^, here named eastern and western Central Asia, can be designated within Central Asia (see Fig. 1). *Nitraria* also occurs in Siberia, generally in Siberian steppe, which is distinct from the Central Asian desert. Thus, five areas are divided for *Nitraria*, **A**: eastern Central Asia, also including North China-Northeast China of East Asia, **B**: western Central Asia, as well as Iran, Turkey, and Eastern Europe, **C**: Siberian steppe, **D**: Africa, northern Africa and adjacent regions of the Middle East, **E**: Australia, see Fig. 1.

Ancestral area reconstructions (AAR) were conducted using both the Statistical Dispersal-Vicariance (S-DIVA) approach and likelihood analysis under the dispersal-extinction-cladogenesis (DEC) model. Analyses were implemented in RASP v2.1 [http://mnhscueducn/S-diva/] and Lagrange [http://reelab.net/lagrange/], respectively.

### Diversification rate

To visualize temporal variation in the diversification rates of *Nitraria*, semi-logarithmic lineage-through-time (LTT) plots were constructed in the R package APE v2.5-1^59^. To evaluate 95% credibility intervals of the empirical LTT curves, 1000 ultrametric trees randomly sampled from the converged BEAST trees were also used to calculate semi-logarithmic LTT plots.

Diversification rates were also calculated using whole-clade methods, net diversification rates^60^. Net rate of diversification (r) of *Nitraria* and some internal clades of interest were calculated following the equation (6) of Magallón and Sanderson^60^ under two extremes of the relative extinction rate (ε = 0.0 and 0.9, respectively).

### Chromosome evolution

The program chromEvol v1.3 (http://www.tau.ac.il/~itaymay/cp/chromEvol/) was used to infer ancestral chromosome numbers in *Nitraria*. The BEAST tree was used as input. The program was run under the default parameters using the best-fitting model selected according to the likelihood ratio tests using the Akaike information criterion (AIC).

## Additional Information

**How to cite this article**: Zhang, M.-L. *et al*. Young dispersal of xerophil *Nitraria* lineages in intercontinental disjunctions of the Old World. *Sci. Rep*. **5**, 13840; doi: 10.1038/srep13840 (2015).

## Supplementary Material

Supplementary S1 Data

Supplementary S2 Data

Supplementary S3 Data
