# Supplementary material for: Young dispersal of xerophil Nitraria lineages in intercontinental disjunctions of the Old World
Source: Sci Rep. 2015 Sep 7;5:13840. doi: 10.1038/srep13840 (PMC4561381; doi:10.1038/srep13840)
Supplement: Supplementary S1 Data [file srep13840-s1.pdf]

Young dispersal of xerophil *Nitraria* lineages in intercontinental disjunctions of the Old World

Ming-Li Zhang, Kamshat Temirbayeva, Stewart C. Sanderson, and Xi Chen

S1: *rbc*L and 7-gene sequence data

***rbc*L sequence data:**

Meliaceae

Trichilia emetica Vahl AY128244

Nymania capensis Lindb. AY128238

Swietenia macrophylla King AY128241

Simaroubaceae

Simarouba glauca DC. AY128252

Ailanthus altissima (Mill.) Swingle AY128247

Rutaceae

Correa pulchella J.B. Mackay ex Sweet AF066816

Chorilaena quercifolia Endl. AF066810

Phebalium woombye (Bailey) Domin AF066822

Diplolaena dampieri Desf. AF066807

Sarcomelicope simplicifolia (Endl.) T.G. Hartley AF066817

Melicope ternata J.R. Forst. & G. Forst. AF116271

Lunasia amara Blanco AF066814

Skimmia anquetilia N.P. Taylor & H.K. Airy Shaw AF066818

Dictamnus sp. AF066801

Casimiroa edulis La Llave AF066808

Choisya mollis Standley AF066800

Pilocarpus pennatifolius Lem. AF066809

Calodendrum capensis Thunb. AF066805

Adenandra uniflora Willd. AF066803

Phellodendron amurense Rupr. AF066804

Zanthoxylum monophyllum (Lam.) P. Wilson ZMU39282

Pleiospermium alatum Wight & Arn. AF066821

Severinia buxifolia (Poir.) Tenore AF066806

Atalantia ceylanica (Arn.) Oliver AF066812

Clausena excavata Burm. f. AF066813

Aegle marmelos (L.) Correa ex Roxb. AF066811

Ruta graveolens L. AY128251

Spathelia excelsa Krause AF066798

Ptaeroxylon obliquum (Thunb.) Radlk. AF123276

Cneorum pulverulentum Vent. U38858

Sapindaceae

Cupaniopsis anacardioides (A. Rich.) Radlk. L13182

Koelreuteria paniculata Laxm. U39283

Acer saccharum L. L01881

Aesculus pavia Castigl. U39277

Burseraceae

Bursera inaguensis Britton L01890

Anacardiaceae

Pistacia vera L. AJ235786

Rhus copallina L. U00440

Schinus molle L. U39270

Biebersteiniaceae

Biebersteinia heterostemon Maxim. DQ408667

Biebersteinia multifida DC. DQ408665

Biebersteinia odora Stephan DQ408666

Biebersteinia orphanidis Boiss. AF035920

Bixaceae

Bixa orellana L. Y15139

Malvaceae

Gossypium hirsutum Cav. M77700

Bombax buonopozense P. Beauv. AF022118

Ochroma pyramidale (Cav. ex Lam.) Urb. AJ233118

Cistaceae

Cistus revolii H.J. Coste & Soulie Y15140

Helianthemum grandiflorum DC. Y15141

Dipterocarpaceae

Anisoptera marginata Korth. Y15144

Muntingiaceae

Muntingera calabra L. Y15146

ITS trnL-trnF rbcL rpS16 psbA-trnH psbB-psbH rpS16-trnK sequence data:

Nitrariaceae

Nitraria schoberi L. KP087771 KP087727 KP087789 KP087747 KP087759 KP087735 KP087779

Nitraria sibirica Pall. DQ267178 DQ267168 DQ267160 KP087748 KP087760 KP087736 KP087780

Nitraria sphaerocarpa Maxim.DQ267177 DQ267167 DQ267159 KP087749 KP087761 KP087737 KP087781

Nitraria roborowskii Kom. DQ309042 DQ267169 DQ267161 KP087750 KP087762 KP087738 KP087782

Nitraria tangutorum Bobr. DQ267176 DQ267166 DQ267158 KP087751 KP087763 KP087739 KP087783

Nitraria retusa Aschers. KP087772 KP087728 KP087790 KP087752 KP087764 KP087740 KP087784

Nitraria retusa Aschers. KP087773 KP087729 KP087791 KP087753 KP087765 KP087741 KP087785

Nitraria komarovii Ili et Lav. KP087774 KP087730 KP087792 KP087754 KP087766 KP087742 KP087786

Nitraria billardieri D. C. KP087775 KP087731 KT377258 KP087755 KP087767 KP087743 KT377261

Peganum harmala L. KP087776 KP087732 KP087793 KP087756 KP087768 KP087744 KP087787

Peganum harmala L. 1 KT377256 KT377267 KT377259 KT377254 KT377264 KT377252 KT377262

Peganum nigellastrum Bunge KT377257 KT377268 KT377260 KT377255 KT377265 KT377253 KT377263

Tribulus terrestris L. KP087777 KP087733 KP087794 KP087757 KP087769 KP087745 KT377266

Zygophyllum xanthoxylum Maxim. KP087778 KP087734 KP087795 KP087758 KP087770 KP087746 KP087788
