# Supplementary material for: Young dispersal of xerophil Nitraria lineages in intercontinental disjunctions of the Old World
Source: Sci Rep. 2015 Sep 7;5:13840. doi: 10.1038/srep13840 (PMC4561381; doi:10.1038/srep13840)
Supplement: Supplementary S2 Data [file srep13840-s2.pdf]

Young dispersal of xerophil *Nitraria* lineages in intercontinental disjunctions of the Old World

Ming-Li Zhang, Kamshat Temirbayeva, Stewart C. Sanderson, and Xi Chen

S2: *rbc*L phylogenetic tree and dating chronogram.

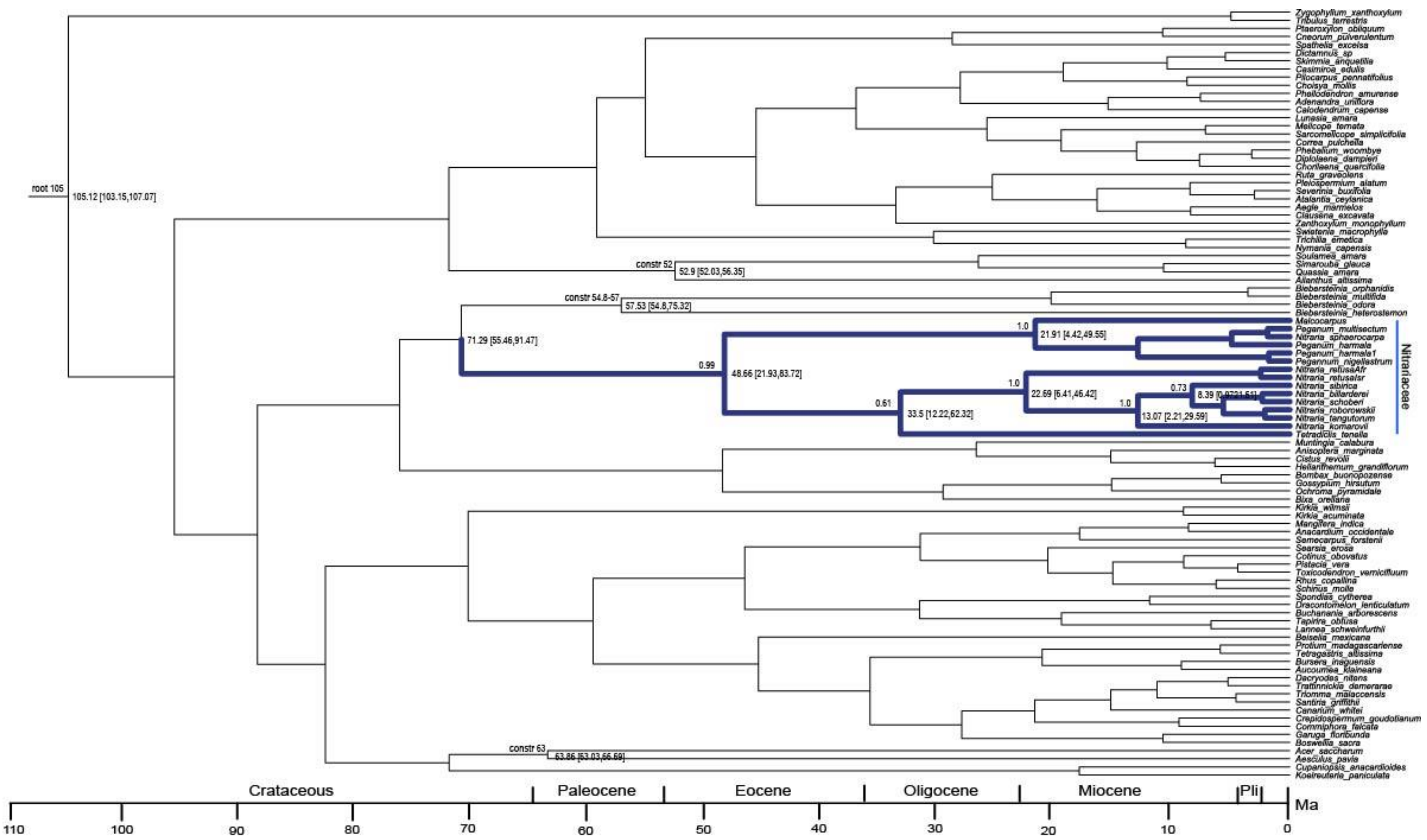

root 105
105.12 [103.15,107.07]
constr 52
52.9 [52.03,56.35]
constr 54.8-57
57.53 [54.8,75.32]
71.29 [55.46,91.47]
0.99
48.66 [21.93,83.72]
1.0
21.91 [4.42,49.55]
1.0
22.69 [6.41,46.42]
0.61
33.5 [12.22,62.32]
1.0
13.07 [2.21,29.59]
0.73
constr 63
63.86 [53.03,56.59]
Zygophyllum_xanthoxylum
Tribulus_terrestris
Ptaeroxylon_obliquum
Cneorum_pulverulentum
Spathelia_excelsa
Dictamnus_sp
Skimmia_anquetilia
Casimiroa_edulis
Pilocarpus_pennatifolius
Choisya_mollis
Phellodendron_amurense
Adenandra_uniflora
Calodendrum_capense
Lunasia_amara
Melicope_ternata
Sarcomelicope_simplicifolia
Correa_pulchella
Phebalium_woombye
Diplolaena_dampieri
Chorilaena_quercifolia
Ruta_graveolens
Pleiospermium_alatum
Severinia_buxifolia
Atalantia_ceylanica
Aegle_marmelos
Clausena_excavata
Zanthoxylum_monophyllum
Swietenia_macrophylla
Trichilia_emetica
Nymania_capensis
Soulamea_amara
Simarouba_glauca
Quassia_amara
Ailanthus_altissima
Biebersteinia_orphanidis
Biebersteinia_multifida
Biebersteinia_odora
Biebersteinia_heterostemon
Malacocarpus
Peganum_multisectum
Nitraria_sphaerocarpa
Peganum_harmala
Peganum_harmala1
Pegannum_nigellastrum
Nitraria_retusaAfr
Nitraria_retusaIsr
Nitraria_sibirica
Nitraria_billarderei
Nitraria_schoberi
Nitraria_roborowskii
Nitraria_tangutorum
Nitraria_komarovii
Tetradiclis_tenella
Muntingia_calabura
Anisoptera_marginata
Cistus_revolii
Helianthemum_grandiflorum
Bombax_buonopozense
Gossypium_hirsutum
Ochroma_pyramidale
Bixa_orellana
Kirkia_wilmsii
Kirkia_acuminata
Mangifera_indica
Anacardium_occidentale
Semecarpus_forstenii
Searsia_erosa
Cotinus_obovatus
Pistacia_vera
Toxicodendron_vernicifluum
Rhus_copallina
Schinus_molle
Spondias_cytherea
Dracontomelon_lenticulatum
Buchanania_arborescens
Tapirira_obtusa
Lannea_schweinfurthii
Beiselia_mexicana
Protium_madagascariense
Tetragastris_altissima
Bursera_inaguensis
Aucoumea_klaineana
Dacryodes_nitens
Trattinnickia_demerarae
Triomma_malaccensis
Santiria_griffithii
Canarium_whitei
Crepidospermum_goudotianum
Commiphora_falcata
Garuga_floribunda
Boswellia_sacra
Acer_saccharum
Aesculus_pavia
Cupaniopsis_anacardioides
Koelreuteria_paniculata
Nitrariaceae
Crataceous
Paleocene
Eocene
Oligocene
Miocene
Pli
Ma
110
100
90
80
70
60
50
40
30
20
10
0
