# Supplementary material for: Young dispersal of xerophil Nitraria lineages in intercontinental disjunctions of the Old World
Source: Sci Rep. 2015 Sep 7;5:13840. doi: 10.1038/srep13840 (PMC4561381; doi:10.1038/srep13840)
Supplement: Supplementary S3 Data [file srep13840-s3.pdf]

Young dispersal of xerophil *Nitraria* lineages in intercontinental disjunctions of the Old World

Ming-Li Zhang, Kamshat Temirbayeva, Stewart C. Sanderson, and Xi Chen

S3: The average diversification rates estimated for the crown of nodes within *Nitraria*

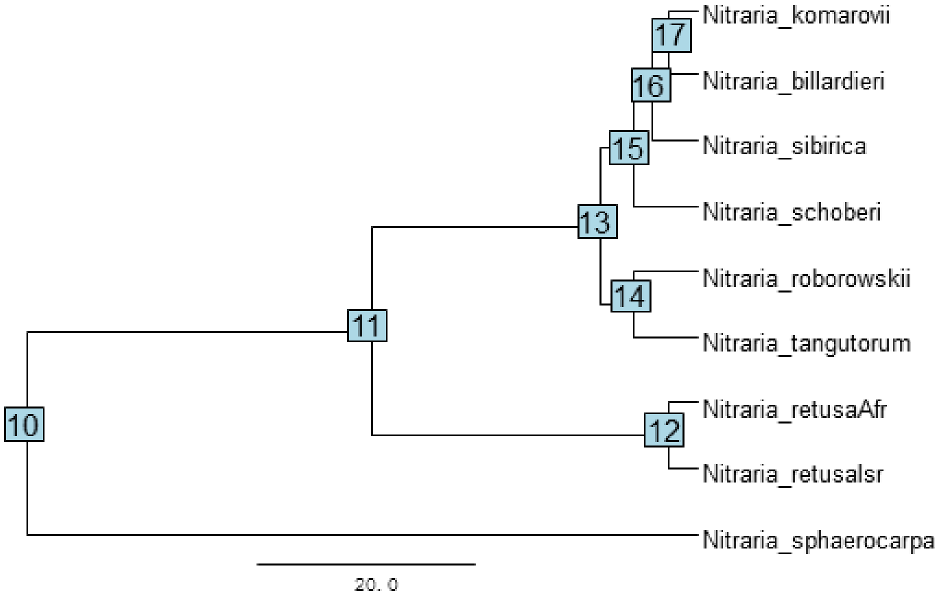

node	Stem age	div	eps=0	eps=0.9
10	61.84	9	0.035530798	0.009505
11	30	8	0.069314718	0.017688
13	8.96	6	0.199973155	0.045253
15	5.95	4	0.232990649	0.044095
16	4.19	3	0.262198637	0.043513
